# Understanding education policy preferences: Survey experiments with policymakers in 35 developing countries

**DOI:** 10.1016/j.worlddev.2025.107140

**Published:** 2025-12

**Authors:** Lee Crawfurd, Susannah Hares, Ana Minardi, Justin Sandefur

**Affiliations:** Center for Global Development, United Kingdom

**Keywords:** Foundational literacy, Vocational education, Bureaucracy, Policy preferences, Conjoint, Discrete choice

## Abstract

Despite rapid increases in global access to primary school, average learning outcomes in many low and middle income countries remain low. International actors are increasingly focused on a policy agenda prioritizing foundational learning, measured by test scores in primary school. However, international actors have limited capacity to impose this agenda, which ultimately depends on national decision-makers. In this paper we present new evidence on the priorities and views of these national decision-makers. We report on a new survey of 931 senior government officials working on education in 35 low- and middle-income countries. We show with survey experiments that national policymakers place relatively low value on action to address foundational skills. We explain variation in preferences among policymakers as a function of three possible factors: different objectives for education (e.g., learning versus socialization), different beliefs about the state of the world (e.g., enrollment and learning levels), and different beliefs about the effectiveness of specific interventions. Misalignment with donor agendas is evident in all three dimensions. We also show experimentally that beliefs do respond to new evidence on the effectiveness of interventions.

## Highlights

• Policymakers prioritize vocational education and socialization over foundational learning.
• This is in part because they overestimate learning levels.
• Policymakers do update in response to new evidence from similar contexts and large-scale studies.
• Policy focus on vocational education is linked to objectives such youth unemployment.

## 1 Introduction

While educational policy is largely driven by domestic political incentives, international norms have clearly contributed to a widespread global expansion in schooling. Primary school enrollment has increased dramatically over the last 50 years, but learning outcomes in many developing countries remain low (Angrist et al., 2021). In order to address this ‘learning crisis’ in the developing world, international organizations including the World Bank and UN are increasingly focusing their education policy agenda on foundational literacy and numeracy. This agenda prioritizes improvements in somewhat narrow measures of literacy and numeracy over broader educational goals; and implies investments in primary over secondary, vocational, or tertiary education; accountability reforms over additional resources for education; and technocratic administration of scientifically proven policies over participatory, local decision-making.^1^ International actors have little ability to enact this policy agenda directly, as foreign aid is a small share of education budgets. Instead, the promulgation of this reform agenda hinges on the diffusion of ideas. Governments in the developing world must be persuaded these policy priorities make sense. Ultimately for outside policy reform efforts to have any chance of success, donors need to make more effort to understand the beliefs of policymakers in order to influence them (Smets, 2019).

In this paper we do three things. First, we ask whether senior bureaucrats in low and middle income countries buy into the new consensus of global elites. Though social scientists usually model bureaucrats as merely the agents of their political principals (Moe, 1984), information asymmetries mean that civil servants can play an important role in shaping the choice set available to elected politicians and constraining their actions (Gailmard and Patty, 2012, Harris et al., 2020). Civil servants have particularly strong influence in imperfect democracies (Ricks, 2018), and in the policy areas that are least politically salient (Baekgaard et al., 2015). There is significant variation across countries in the degree to which bureaucratic structures are based on Weberian principles of neutral merit-based recruitment vs political appointment (Dahlström et al., 2012). We report on a survey of 931 senior officials from 35 developing country governments, primarily from ministries of education and related agencies. We find that national policymakers place a higher priority on vocational education than foundational learning. We show this both through direct survey questions, and indirectly through a ‘conjoint experiment’ in which respondents choose between specific hypothetical concrete projects with different randomized features. Our work here complements other recent studies using conjoint or discrete choice experiments to study preferences over alternative social policies in the developing world (Briggs, 2021, Kim et al., 2025, Redfern et al., 2019, Solomon and Zeitlin, 2019), which has typically been more common in the health sector (Mirelman et al., 2012), and research understanding how policymakers value different social policies (Toma & Bell, 2024).

Second, we seek to understand the reasons for the divergence in preferences between national policymakers and global elites. One potential reason for differences in their preferences are different emphases on the function of education. International policymakers focus primarily on the role of education in building human capital and economic inclusion. By contrast for national policymakers education plays a critical role in ‘socialization’, as recognized by Emile Durkheim in his foundational sociology of education. Recent evidence shows how government investments in universal schooling have been motivated by nation-building — with the supply of universal schooling predating in most countries any democratic demand for it by decades (Paglayan, 2021). These is a wide literature on the role of education in shaping political views, all the way from Durkheim (1922) through to more recent empirical research demonstrating causal effects on political views (Cantoni et al., 2017). There are many potential objectives of an education system, from building literacy and numeracy skills, to socialization, to development of non-cognitive skills or creativity. We confront policymakers with a trade-off, using another conjoint experiment to allow officials to choose between alternate outcomes, again with randomized features. We find that policymakers rank ‘socialisation’ as the highest priority outcome of the education system, followed by secondary school completion, with foundational literacy and numeracy last (in contrast to the emerging focus of global elites). Other reasons for differences in preferences might be differences in beliefs about the scale of policy problems and the availability of effective interventions. We show that policymakers significantly over-estimate foundational learning levels in their country (and therefore underestimate the scale of the policy problem). Specifically, policymakers guess that on average 47 percent of 10 year olds in their country can read, compared with World Bank estimates based on actual assessment data of just 23 percent. This contrasts with much more accurate beliefs about other more visible features of school systems; such as enrollment levels, government spending, and labor market benefits. Here our study relates to other work understanding the relevant knowledge of public officials (Bold et al., 2017, Das et al., 2016, Liu et al., 2017, Rogger and Somani, 2024). We find that individual preferences for spending on foundational learning are correlated with their beliefs about actual learning levels. Foundational skills may thus be under-prioritized in part because policymakers do not realize how bad the situation really is. Policymakers do seem to recognize the importance of education in the labor market, and perceive there to be effective interventions. We elicit policymakers’ perceptions about the labor market returns to education in a similar fashion to Jensen (2010). Policymakers believe that pecuniary returns to secondary schooling are high, particularly for poorer pupils. This contrasts somewhat with a common view of education systems as primarily playing a sorting role or providing a “filtration system” designed to select the most talented individuals for further education and eventual administrative jobs (Muralidharan & Singh, 2021).

Finally, we find that officials *are* receptive to empirical evidence on “what works” in education. We conduct a survey experiment which randomly (but truthfully) varies the description of real empirical research results. We measure the prior and posterior beliefs of officials about effect sizes. We find that officials place no weight, or even negative weight, on randomized experiments per se. They are more positively influenced by sample size and contextual similarity of the study setting to their own country. Here we add to an emerging literature on how policymakers respond to evidence. Though government officials are subject to cognitive biases in decision-making (Banuri et al., 2019, Vivalt and Coville, 2023), they do update their beliefs in response to new information (Lee, 2022, Masset et al., 2013) and follow through on different policy actions (Hjort et al., 2021). Our findings are in line with others that find that policymakers from multilateral development banks (Vivalt et al., 2025) and US education agencies (Nakajima, 2021) care more about external validity than internal validity. Our paper joins a growing body of work in social science conducting experiments with political elites (Kertzer & Renshon, 2022) and bureaucrats (Kalaj et al., 2022).

The rest of the paper is organized as follows. In Section 2 we present the survey design and respondent characteristics. In Section 3 we present estimates of policy preferences of policymakers. In Section 4 we set out potential explanations for variation in policymaker preferences. Section 5 concludes.

## 2 Data

Our sample frame is all senior government staff with an education or aid related job, now or in the recent past. The modal respondent is a Director in a Ministry of Education. This is a senior civil service management position, typically with just one or two civil servant positions above them in the organizational hierarchy (such as a Director-General and/or Permanent Secretary). Directors are in some countries political appointees, and in others are non-political career civil servants. Our full sample spans from some Ministers (political leaders) to some more junior officers, at Ministries of Finance, and independent agencies for technical or higher education. In each country we recruited a consultant with good networks and access who first compiled a draft list of potentially relevant senior officials. This list was reviewed by the research team, and consultants then conducted interviews, in-person where possible or by phone (many countries had mobility restrictions in place due to COVID-19). The initial set of lists contained 1,056 potential respondents. Overall 684 of these potential respondents were successfully interviewed (65 percent). An additional 247 interviews were conducted with respondents who were not on the initial lists compiled by consultants, but did meet the criteria for interview, for a total of 931 interviews.^2^ Surveys began on 5th March 2020 and continued through 9th September 2020.

### 2.1 Country sample

We selected countries purposefully to cover a range of income levels and geographies, with a weighting toward those with weak educational outcomes and high levels of aid for education (based on OECD Creditor Reporting System data). Selection was also influenced through practical considerations in terms of the availability of consultants with the required access to respondents. The majority of these countries are low income (18) or lower-middle income (13) according to World Bank classifications. Twelve are in Anglophone Africa, 12 in Francophone Africa, and 6 in Asia-Pacific (Table 1, Figure A4).

**Table 1:** Country Sample (Region and income group).

	Anglophone Africa	Francophone Africa	Asia-Pacific	Other	Total
LIC	Gambia, Liberia Malawi, Sierra Leone Tanzania, Uganda	Benin, Burkina Faso DRC, Madagascar Mali, Rwanda, Togo	Nepal	Guinea-Bissau Haiti Mozambique Somalia	18
LMIC	Ghana, Kenya Nigeria, Zambia	Cameroon, Comoros, Congo, Cote d’Ivoire	Bangladesh Pakistan Solomon Islands Vanuatu	Angola	13
UMIC	Namibia, South Africa	Gabon	Georgia		4
Total	12	12	6	5	35

Note: LIC stands for low-income countries, LMIC for lower-middle income, and UMIC for upper-middle income, all according to the World Bank’s country income classification.

### 2.2 Characteristics of policymakers

Most officials in our sample are middle-aged men. Seventy-two percent are male, and have a median of 11 years of experience. Forty-two percent are Directors or Director Generals, and 22 percent Deputy or Assistant Directors. The sample included 29 current, former, deputy, and sub-national Ministers of Education. Fifty-seven percent of officials work for a Ministry of Education, nine percent in an independent technical and vocational (TVET) or skills agency, eight percent in an independent higher education agency, and three percent in the center of government (Ministry of Finance, President’s Office, or Planning Commission). Forty-one percent of officials are from Anglophone African countries, 28 percent from Francophone Africa, and 17 percent from Asia (Table 2).

**Table 2:** Characteristics of officials .

	Full	Asia-	Anglo-	Franco-	Others*
	Sample	Pacific	phone	phone	
			Africa	Africa	
Agency (% of respondents)					

Ministry of Education	66.7	68.9	66.1	61.8	76.0
TVET/Skills ministry/agency	8.2	3.1	8.9	14.1	0.0
Higher Education Ministry/Agency	6.3	1.9	8.1	7.3	4.8
Centre of government	6.3	15.5	4.4	3.8	5.6
University	4.3	3.1	2.1	7.3	6.4
Local government	2.5	0.6	5.7	0.0	0.0
Others	1.9	1.2	3.1	1.5	0.0
Missing	3.8	5.6	1.6	4.2	7.2
Total	100.0	100.0	100.0	100.0	100.0

Job title (% of respondents)					

Minister	2.4	5.6	1.3	0.0	6.4
Advisor	3.5	0.0	0.5	6.1	12.0
Permanent secretary/Director general	9.0	13.0	7.0	12.2	3.2
Director	32.4	24.8	33.1	33.2	38.4
Assistant/Deputy director	23.6	23.0	31.5	17.6	12.8
Officer	17.3	25.5	15.4	18.7	9.6
Academic	4.3	3.1	2.1	7.3	6.4
Missing	7.5	5.0	9.1	5.0	11.2
Total	100.0	100.0	100.0	100.0	100.0

Gender (% of respondents)					

Female	26.6	30.4	31.8	19.1	21.6
Male	71.7	66.5	67.4	79.0	76.0
Missing	1.7	3.1	0.8	1.9	2.4
Total	100.0	100.0	100.0	100.0	100.0

Region (Row Percentage)	100.00	17.31	41.09	28.17	13.44

Observations	931	161	383	262	125

Note: *Others includes Lusophone Africa (Angola, Mozambique & Guinea-Bissau), Haiti and Somalia. Centre of Government includes officials based at the President or Prime Minister’s Office, or Ministry of Finance, Planning, or Public Service.

## 3 Measuring policymaker preferences

We measure policymaker policy preferences both directly and indirectly. We first ask officials to choose a topic for a hypothetical aid project. The most common response is technical and vocational education (54 percent of respondents – Figure A5). We also ask them to prioritize indicators from the Sustainable Development Goals. The indicators related to TVET (SDG 4.3) and employment skills (SDG 4.4) are ranked more highly than foundational learning (Figure A6). We also ask what officials view to be the most important reform in the last five years. The most common responses are curriculum and free education (mentioned by 20 percent of respondents each). Technical and Vocational education (TVET) is the fourth most frequently mentioned reform (by 10 percent of respondents). A reading or literacy program was mentioned by just 2 percent of respondents A7).

Aid is not an open marketplace — recipients are unlikely to reject a project or push back too strongly on resource decisions made by donors. We therefore use a conjoint experiment to allow (and force) respondents to make an explicit choice between two concrete options, allowing us to draw out and estimate underlying preferences. Conjoint experiments can mitigate social desirability bias, because respondents are not asked to make absolute judgments or state preferences directly. Instead, they choose between multi-attribute profiles, making it harder to infer which specific attribute they are prioritizing, and thus reducing pressure to conform to socially acceptable views (Horiuchi et al., 2021). The forced comparison also gives us a quantitative estimate of the dollar value respondents place on projects in different sectors. We ask officials to choose between two hypothetical aid projects. Each respondent makes six binary choices between two projects. Each project has three attributes that are randomly generated for each choice (Figure A8);

(1) the focus of the project (information technology, school construction, foundational literacy, assessment, or technical and vocational education),

(2) the total dollar budget of the project ($30 m, $32 m, $34 m, $36 m, $38 m, or $40 m) and

(3) whether the project comes with one, two, or no full-time technical advisors.^3^

Respondents were not explicitly told whether the project would be financed through a loan or a grant, but the wording emphasized the budget as the cost of implementing the project, rather than an amount that would have to be repaid by the government. We therefore interpret this variable as being perceived by respondents as the size of a gift or transfer—akin to a grant to the Ministry of Education or the government more broadly. This framing allows us to interpret the coefficient on the “Budget” variable as an estimate of the marginal value respondents place on additional spending, relative to other project attributes. This allows us to assess trade-offs between financial size and the other features (project focus, and inclusion of technical advisors).

Our analysis of the experimental data is grounded in a random utility model a la McFadden (1973). The respondent chooses the bundle of attributes that gives them the most utility. Therefore, formally project B is chosen over project A by individual i if their utility derived from that project is greater, or if $U_{Bi}>U_{Ai}$. The probability that this will occur is a function of a vector X of three project characteristics, and an individual-specific error term epsilon.

(1) $$P\left[U_{Bi}-U_{Ai}>0\right]=P\left[\overset{3}{\underset{k=1}{\sum }}\beta_{k}\left(X_{Bk}-X_{Ak}\right)+\left(\epsilon_{Bi}-\epsilon_{Ai}\right)>0\right]$$

We therefore regress project choice (A or B) on the characteristics of those two projects. Our results show no substantial difference between Marginal effects from the Logistic model and the Linear Probability Model, hence we present and discuss the results from the latter. The marginal effects of the Logistic model are discussed in the appendix for robustness. We include individual (respondent) fixed effects, so identification comes solely from the randomized differences between the paired projects shown to the same official.

Turning to the results presented in Table 3, each $1 million increase in the budget of a bundle choice increases the probability of that project bundle being chosen by 1.2 percent. This finding in itself is uninteresting, but it allows us a dollar value comparison for other project attributes. Holding budget constant, being offered a TVET project (rather than an IT project) increases the chance of a project being chosen by 13 percent. None of the other project types are statistically significantly different from the omitted base category (IT project). Thus officials are roughly indifferent between $30 million in funding for a TVET project and $40 million for a project on assessment, foundational literacy, school construction, or IT. There is a stronger preference for TVET projects from officials who work in TVET agencies (28 percent more likely to be chosen), but we still see a positive choice for TVET amongst non-TVET agency officials (12 percent more likely to be chosen). Each additional technical advisor on a project causes an increase in a project bundle being chosen by 3.8 percent. Hence, we can infer the value of each additional technical advisor at around $3 million.

**Table 3:** Conjoint experiment results: Aid project preferences.

	Full	TVET Ministry/	Other ministries
	Sample	Agency	& agencies
Budget (USD million)	0.0123***	0.00481	0.0136***
	(0.00182)	(0.00696)	(0.00192)
Technical advisors	0.0384***	0.0913***	0.0317***
	(0.00784)	(0.0329)	(0.00822)
TVET	0.129***	0.282***	0.115***
	(0.0192)	(0.0653)	(0.0205)
Assessment	0.0227	0.128*	0.00674
	(0.0193)	(0.0709)	(0.0206)
Foundational literacy	−0.00534	0.0528	−0.00762
	(0.0193)	(0.0747)	(0.0204)
School construction	0.0223	0.200***	0.00632
	(0.0188)	(0.0715)	(0.0199)
Respondent FE	Yes	Yes	Yes

Obs. (Responses)	8,558	568	7,676
Obs. (Respondents)	733	48	657
R^2^	0.017	0.058	0.016

P-value on tests of equality:			
Assessment = School Construction	0.983	0.340	0.983
Assessment = FL	0.145	0.322	0.481
FL = School Construction	0.145	0.052	0.487

Note: The unit of observation is a hypothetical aid project presented to an individual respondent, and the dependent variable is an indicator that the project was selected as preferable (from a set of two options). Estimates are based on a linear probability model. Results are similar using a logit model (Table B4 and Table B5). The omitted category for projects is an IT project. Results are similar when estimating marginal means rather than average marginal component effects (Leeper et al., 2020). Standard errors, clustered at the paired comparison level, are in parentheses. All specifications include individual fixed effects. * p<0.1, ** p<0.05, *** p<0.01.

## 4 Explaining policymaker preferences

Different policy priorities could be due to different objectives, or different information. Some donor rhetoric stresses the need to concentrate public spending on primary education. This choice is often justified by the role of foundational learning as a long-term investment in human capital, particularly for disadvantaged groups. Despite high enrollment in primary school, basic learning levels in primary school remain very low even in countries rapidly expanding access to higher education tiers. In contrast, national policymakers spend large shares of education budgets on secondary, vocational, and tertiary education, somewhat out of proportion to the share of pupils attaining these levels. This pattern may be attributable to the priority that policymakers place on socialization and political indoctrination over basic literacy and numeracy goals (Durkheim, 1922). Empirical studies show that unemployed youth can lead to political instability and even violence, giving governments good reason to focus on investments that promise to address this issue, such as vocational education (Blattman & Miguel, 2010). Note that the comparison we discuss here reflects observed donor and government priorities rather than advocating for specific spending distributions. In the following sections we present new survey data on policymakers’ perceptions of the objectives of education, the status quo of education in their countries, and the effectiveness of interventions to improve learning.

### 4.1 Different objectives

How do policymakers weight different objectives of education? We conduct a second conjoint experiment in order to estimate these weights. Is education for providing universal basic skills, for getting children through school, or for socialization? When these goals are in tension, what kinds of trade-offs are policymakers willing to make? Preferences over these goals should inform preferences over policy.

We present each official with four binary choices between two hypothetical states of the world. Each state has three education outcome attributes that are randomly varied for each choice (Figure A8):

(1) the share of the population with foundational literacy (40, 60, 80, or 100 percent),

(2) the share completing secondary school (40, 60, 80, or 100 percent), and

(3) the share that are ‘dutiful citizens’ (70, 80, 90, or 100 percent).

We use the notion ‘dutiful citizens’ as a neutral description of the outcome of an education system that prioritizes socialization and nation-building. Whilst we should expect differences in how this phrases is interpreted across countries, our analysis is focused on different choices within individuals, so we can abstract from these differences in interpretation. We ask respondents which state of the world they would prefer between two hypothetical scenarios.

We estimate Eq. (1) presented in Section 3 using the education outcomes presented above as the attributes. The results from this experiment show that officials place a somewhat higher value on dutiful citizens than they place on foundational learning or on completing secondary school. Specifically, an education system that generates ten percentage points more dutiful citizens makes an official ten percent more likely to choose it, whereas a system that generates ten percentage points more children with foundational literacy makes an official only seven percent more likely to choose it (Table 4). In other words, dutiful citizens are worth nearly 50 percent more to officials than children learning how to read.

**Table 4:** Conjoint experiment results: Education outcomes preferences.

	Full	TVET	Other
	Sample	Ministry/	Ministries
		Agency	& agencies
＋10% point with FL	0.0656***	0.0444***	0.0686***
	(0.00414)	(0.0146)	(0.00440)
＋10% point complete secondary	0.0774***	0.0895***	0.0764***
	(0.00424)	(0.0154)	(0.00452)
＋10% point are dutiful citizens	0.0974***	0.0848***	0.0966***
	(0.00592)	(0.0211)	(0.00631)
Respondent FE	Yes	Yes	Yes

Obs. (Responses)	6,730	542	5,942
Obs. (Respondents)	853	68	753
R^2^	0.145	0.139	0.147

P-value on tests of equality:			
Literacy = Secondary School	0.111	0.056	0.360
Citizen = Secondary School	0.010	0.871	0.015
Citizen = Literacy	0.000	0.095	0.001

Note: The unit of observation is a hypothetical state of the world, presented to an individual respondent. The dependent variable is an indicator that this state of the world was preferred (from a set of two options). Estimates are based on a linear probability model. Results are similar using a logit model (Table B4 and Table B5). At the foot of each column we report p-values on the null that one attribute is as equally valued as another. Standard errors, clustered at the pair-choice level, are in parentheses. * p<0.1, ** p<0.05, *** p<0.01.

### 4.2 Different beliefs about reality

**Beliefs about foundational learning**

**Fig. 1:**
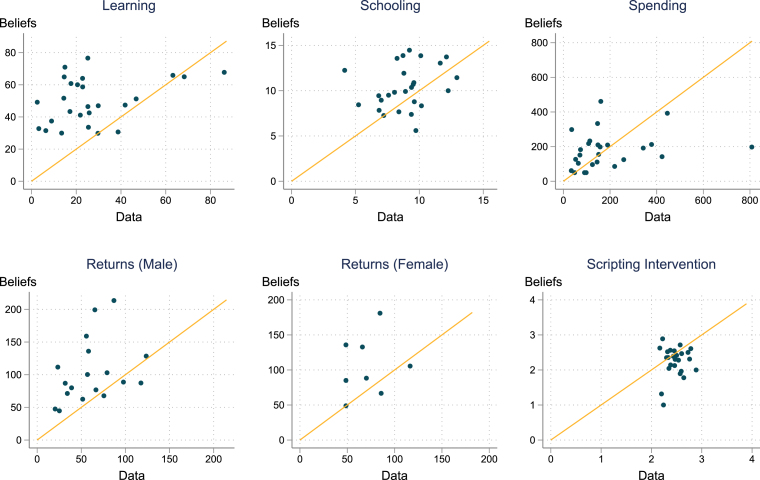
Policymaker Beliefs and Data on Education Systems. Note: This figure shows average responses for each country, compared against data for each country from external sources. Panel (a) Respondents were asked to estimate the share of 10 year olds in their country that have reached the appropriate minimum learning level expected for their age. This is compared with estimates based on the World Bank Learning Poverty indicator. Panels (b) and (c) compare respondent estimates of average schooling and average per pupil spending with data from the World Bank Development Indicators. Panels (d) and (e) compare respondent estimates of the wage gain from secondary school to data-based estimates from Montenegro and Patrinos (2014). Panel (f) compares respondent estimates of the effectiveness of a scripted lessons intervention (on a 0–4 scale) with the actual estimates from one of three studies (Cilliers et al., 2016, Jackson and Makarin, 2018, Piper et al., 2018).

Do policymakers have weak support for foundational learning because they do not recognize that there is a learning crisis? When asked directly, the overwhelming majority of respondents agreed that there is a learning crisis — globally (77 percent) and nationally in their own country (81 percent – Table 5). However officials underestimate the scale of this crisis. We ask them to estimate the share of students in their country that can read by age 10. We then compare this to estimates of the actual shares of students, calculated using the World Bank Learning Poverty indicator.^4^ Officials systematically and in some cases quite dramatically over-estimate the share of pupils who can read at an appropriate level by age 10. Though perceptions are correlated with assessment data, on average officials in our sample estimate that 47 percent of children can read by age 10. This compares to World Bank estimates based on actual national learning assessments for the countries in our sample of just 25 percent (Table 5). By contrast, we see much smaller differences between average beliefs and actual data with regards to average levels of schooling or government spending per pupil (Fig. 1). This over-estimation of reading levels may partially explain the low priority given by national officials to foundational learning.

**Beliefs about potential effect of schooling on intelligence (‘Growth mindset’)**

Can education improve intellectual ability, or is its main function to select or sort the brightest children for further education and elite jobs? Many teachers in developing countries have a “fixed mindset” - believing that “there is little they can do to help a student learn if parents are uneducated” (Sabarwal et al., 2021). If policymakers share such views it might reduce their focus on policy to improve learning. To test this proposition we adopt the three-item growth mindset scale developed by Dweck (2000). Each official is asked on a Likert scale the extent to which they agree or disagree that:

(a) “*You have a certain amount of intelligence, and you can’t really do much to change it*”,
(b) “*People’s intelligence is something that you can’t change very much*”, and
(c) “*People can learn new things, but you can’t really change basic intelligence*”.

Whilst there is some controversy around the use and measurement of the concept of intelligence (Nisbett et al., 2012), empirical evidence does support the view that education causally increases intelligence as measured by IQ tests (Ritchie & Tucker-Drob, 2018).

We find that only around a third of respondents have a ‘growth mindset’, defined as disagreeing with the statement that intelligence is something you cannot change very much. Comparable cross-country data on the growth mindset of adults is not available, however the 2018 PISA survey asks 15-year olds one of these questions across 77 mostly high-income countries. Sixty-three percent of these 15-year olds disagree that ‘*People’s intelligence is something that you can’t change very much*’. Following Dweck (2000) we construct a growth mindset score on a 1–6 scale, which is the simple mean of responses to the three questions. Strongly agree equals a score of 1, agree = 2, disagree = 5, and strongly disagree = 6. We changed the central response categories used by Dweck (3 = mostly agree; 4 = mostly disagree) to instead align with the 5-point Likert scale we use for other questions, and so we score “neither agree or disagree” as 3.5.

**Beliefs about labor market returns to schooling**

Another factor that may explain policymakers’ support for investments in basic education versus secondary or vocational schooling is their perception of the market returns to these different schooling levels.

Do officials have accurate beliefs about the labor market benefits of schooling? Do these beliefs vary by student characteristics? We adopt a similar approach to Jensen (2010). Concretely, we ask respondents what they expect the average earnings to be for a hypothetical child when they are age 30, depending on whether they complete primary school only, or primary and secondary school. Each respondent is asked for these two data points from four hypothetical children. The four children are either high or low intelligence, and from a rich or poor household. The order in which these options are presented is randomly assigned. We randomly assign each respondent to answer about either boys or girls. This allows us to calculate the expected returns to secondary school, and estimate how this varies by the intelligence and family income of the child.

The average policymaker estimate for the labor market returns to secondary schooling are between 93 percent for boys and 100 percent for girls (this difference is *not* statistically significant). The average actual returns based on Mincer regressions with household survey data are 63 percent for boys and 74 percent for girls (Table 5).

**Table 5:** Beliefs about reality.

	Mean (Beliefs)	Mean (Data)	SE (Beliefs)	N (Beliefs)	N (Data)
Global learning crisis (0/1)	.77	.	.42	932	.
National learning crisis (0/1)	.81	.	.39	932	.
10yr olds can read	47.12	25.17	23.19	729	31
Average schooling (Years)	10	9.03	3.7	810	31
Gov spend per child (USD)	177.6	227.42	194.7	624	27
Growth mindset (1–6)	3.55	.	1.6	880	.
LM returns for boys	92.79	61.66	74.56	396	21
LM returns for girls	99.37	76.28	89.68	151	23
Effect of reading program (0–4)	2.31	2.48	.97	884	35

Notes: Data on reading comes from the World Bank reading poverty indicator, and for schooling and spending from the World Bank Development Indicators. Data for labor market returns are drawn from Montenegro and Patrinos (2014). The effects of three reading programs described to respondents come from Cilliers et al. (2016), Jackson and Makarin (2018), and Piper et al. (2018). Further detail about the estimates of labor market returns is contained in Annex D and Table D1.

### 4.3 Beliefs about interventions to improve learning

Finally, support for investments in the quality of basic education may be limited by a perceived lack of effective policy levers. Do policymakers think that it is possible to improve foundational learning? We asked policymakers to estimate the effectiveness of a scripted lesson intervention on student learning. Scripted lessons are one of six ‘good buy’ interventions recommended by a World Bank expert panel to improve learning in low- and middle-income countries (Global Education Evidence Advisory Panel, 2020). Scripted lessons are structured, pre-designed lesson plans that guide teachers step-by-step on what to say and do in the classroom.

We think that most policymakers do not have a strong intuitive sense of what kind of standard deviation effect size is relatively large or small, and thus we avoided talking about standard deviations and instead chose the subjective answers of small, medium, large, very large, or no effect. Whilst respondents might have different interpretations of what these mean, randomization of treatment across respondents means that different randomized groups should on average have similar interpretations, and so this does not bias our results. For each study we asked respondents if they thought it would have a small, medium, large, very large, or no effect. We score a response of no effect as 0, small effect as 1, medium effect as 2, large effect as 3, and very large effect as 4. On this zero to four scale the mean response was 2.3 (a medium effect), close to the actual mean effect across the three studies of 2.48.

We chose to use these subjective responses rather than asking policymakers to estimate standard deviation effect sizes, as we do not expect most policymakers to have a strong intuitive sense of what kind of effect size is relatively large or small. Whilst respondents likely do have different interpretations of what a small or large effect is, randomization of treatment across respondents means that different randomized groups should on average have similar interpretations, and so this does not bias our results.

Each policymaker was asked to estimate the effect of one of three randomly selected studies that evaluate interventions providing detailed lesson guides (‘scripted lessons’) for teachers. All three studies are randomized control trials. One study involved 50 schools in South Africa (Cilliers et al., 2016), one involved 170 schools in the United States (Jackson & Makarin, 2018), and one involved 800 schools in Kenya (Piper et al., 2018). We translate effect sizes using the benchmarks reported by Kraft (2020). Therefore we classify the effects in South Africa (0.12 σ) and the United States (0.06–0.09 σ) to be *medium* effect sizes, and the effect sizes in Kenya (0.38–1.29 σ) as being *very large*.

### 4.4 Explaining variation in policymaker preferences

We have discussed three categories of explanation for policymakers’ low preference for investments in foundational learning. How important are these potential explanations? In this section we present regression estimates of the correlates of individual policymaker preferences.

Our outcomes of interest are indices for the strength of policymaker preference for foundational learning and for TVET. The foundational learning preference is constructed using questions on sectoral priorities for new aid (Figure A5), and on the ranking of Sustainable Development Goals (Figure A6). The TVET preference is constructed using the same two questions, counting responses for TVET, employment skills, or skills for sustainable development as a preference for TVET.^5^

We then regress policy preferences on beliefs about reality (foundational learning levels and average schooling), about returns to interventions (labor market returns to schooling, growth mindset, and a scripted lessons interventions), and individual characteristics (Table 6.

A one standard deviation increase in beliefs about foundational learning levels is correlated with a lower preference for foundational learning (0.1 σ) and a higher preference for TVET (0.14 σ). There is no statistically significant correlation between preferences and beliefs about average schooling, about the labor market returns to schooling, about growth mindset, or about the effectiveness of scripted lessons. Men have a lower preferences for foundational learning than women (0.16 σ), and officials who work in a dedicated TVET agency have a much stronger preference for TVET (0.65 σ). One theory is that preferences for marginal aid spending may reflect the existing distribution of aid spending. To test this, we calculate the share of education aid spending in each country that goes on TVET, based on data from the OECD Creditor Reporting System (CRS). The share of education aid to TVET in the countries in our sample varies between two percent in Angola to 38 percent in Rwanda. There is a positive but statistically insignificant correlation between this share and preferences for further TVET spending. Countries with higher university graduate unemployment rates have *lower* preferences for both TVET and foundational learning. One interpretation is that in countries with high graduate unemployment officials might prefer to channel scarce resources towards universities to improve quality, crowding out spending on both TVET and basic skills. Another possibility is reverse causation: governments that have historically under-invested in foundational skills and vocational pathways end up producing graduates ill-matched to labor-market demand, yielding both high graduate joblessness and the observed preference pattern.

**Table 6:** Explaining spending preferences.

	FLN		TVET	
	(1)	(2)	(3)	(4)
*Beliefs about reality:*				
Percent of 10-year-olds who can read (z-score)	−0.0842*	−0.0705	0.136**	0.145**
	(0.0438)	(0.0440)	(0.0569)	(0.0628)
Average schooling (years)	−0.00136	0.00127	0.00144	−0.0155
	(0.0151)	(0.0169)	(0.0209)	(0.0226)
LM Returns (z-score)	0.0253	0.0241	0.0236	0.00974
	(0.0332)	(0.0360)	(0.0532)	(0.0548)
Growth Mindset (1-6 score)	0.0124	0.00255	0.0435	0.0538
	(0.0301)	(0.0264)	(0.0503)	(0.0456)

*Beliefs about interventions:*				
Scripted lessons (0–4 scale)	0.0591	−0.0302	−0.0713	−0.0187
	(0.0616)	(0.0513)	(0.0648)	(0.0471)

*Respondent Characteristics:*				
Male		−0.191**		0.0411
		(0.0723)		(0.0800)
TVET/Skills Ministry/Agency		−0.129		0.616***
		(0.174)		(0.185)

*Country Characteristics:*				
Share of aid on TVET		0.290		0.808
		(0.473)		(1.044)
Graduate Unemployment Rate		−0.0206**		−0.0259*
		(0.00840)		(0.0140)
Region FE	Yes	Yes	Yes	Yes

Obs.	786	672	786	672

R^2^	0.02	0.05	0.03	0.11

Note: The outcome variable is an index summarizing the strength of individual preference for foundational learning or for TVET. We show results for the individual components of this index in Table B2 and Table B3. Standard errors are clustered at the country level. * p<0.1, ** p<0.05, *** p<0.01.

## 5 Experimental evidence on changing beliefs

Do research findings change people’s minds? In this section we report on the results of the information experiment involving scripted lessons mentioned earlier. In this experiment we seek to understand how and whether research findings influence policy views.

We use an identical vignette describing the study set up, but randomly vary the study details that are revealed — specifically the country that the study was conducted in, the number of schools involved in the study, and whether we mention that the study was a randomized control trial or not. Officials were first asked for their prior on the effect size of the study. We then provide evidence on the actual effect of the study. After revealing what the effect size actually was, we estimate posterior beliefs by asking the official what they think the effect size would be if the project was replicated in their country. We then calculate whether officials update their beliefs towards the true value, as the difference between (a) the absolute gap between the true effect and the posterior belief, and (b) the absolute gap between the true effect and the prior. For example, someone who was presented the South Africa study (with a medium effect size, or 2 points) and whose prior was a large effect (3 points) would have a prior gap of one point. If after being presented the true effect they updated their estimate for a replication in their context to be a medium effect (2 points), the gap would have reduced by one point, and they are classed as having updated towards the evidence presented.

(2) Update=Posterior−Prior

Thirty-one percent of officials correctly estimate the true effect size of the study that they are presented with. Sixty-four percent of officials do not change their belief after receiving the new information at all. Fifteen percent update their belief in the direction of the evidence presented. Twenty-two percent *increase* the gap between their prior estimate and the true value. We create a binary indicator for whether someone updated their belief, and estimate the following equation with a linear OLS regression:

(3) $$Update=\beta_{1}\text{RCT}+\beta_{2}\text{Study Size}+\beta_{3}\text{DevelopingCountry}+\epsilon$$

Studies from a developing country (South Africa or Kenya) increased the chance of the official updating their beliefs towards the effect found in the study, by around five percentage points (Table 7). This is consistent with our expectation that evidence from a similar low- or middle-income country context is considered more relevant than evidence from a high-income country. Revealing that the study was an RCT has no effect on the probability of updating beliefs towards the true effect found in the study. The coefficient is negative, and we are able to rule out positive effects of larger than two percentage points. A study that is 100 schools larger increases the chance of belief updating, by two percentage points. These results support the notion that evidence from a relevant context is more likely to change minds than evidence from a randomized control trial in a less relevant context, and that larger studies are more likely to change minds. One possibility is that officials in countries where more RCTs have been conducted might be more familiar with the value of the method, and more likely to place greater weight on evidence from an RCT. To test this possibility we measure the number of RCTs completed in each country, using data from the American Economic Association’s social science registry (www.socialscienceregistry.com). This varies between zero in Angola, Comores, DRC, Gabon, Haiti, and Vanuatu, to 78 in Kenya. We define a binary indicator for the four countries with 30 or more completed RCTs (Bangladesh, Kenya, Malawi, Uganda), and test the interaction of this indicator with the RCT treatment variable. This interaction term is statistically insignificant.

**Table 7:** Effect of study characteristics on belief updating.

	(1)	(2)	(3)	(4)
	Full	Exc Kenya/SA	Full	Full
*Study Characteristics:*				
Study was RCT	−0.016	−0.012	−0.016	0.004
	(0.023)	(0.023)	(0.023)	(0.028)
N of schools (100s)	0.022***	0.022***	0.022***	0.021***
	(0.004)	(0.005)	(0.004)	(0.005)
Developing Country	0.054**	0.049**	0.054**	0.055**
	(0.023)	(0.023)	(0.023)	(0.023)

*Country Characteristics:*				
rcts30			−0.025	0.008
			(0.035)	(0.050)
treatXrcts30				−0.073
				(0.049)

Obs.	880	852	880	880
R^2^	0.045	0.045	0.046	0.048
Baseline Mean	0.16	0.16	0.16	0.16

Note: The outcome variable is a binary indicator for whether or not the respondent updated their prior belief in the direction of the revealed study effect size. ‘Developing Country’ is a dummy variable with value 1 if the reported study was in Kenya or South Africa and 0 if in the United States. Column (2) excludes officials from Kenya and South Africa as they are the study countries. Standard errors are clustered at the country level.

## 6 Conclusion

In this paper we present new data on the policy preferences of civil servants working on education in low- and middle-income countries. Whilst many global elites increasingly focus on the importance of foundational literacy and numeracy, national civil servants have stronger preferences for technical and vocational education, and for the socialization role of education. These differences in priorities can in part be attributed to a gap in the understanding of officials about the scale of the challenge in foundational literacy. We also show that presenting contextually relevant research evidence to officials can change their beliefs about the effectiveness of an intervention to improve learning.

Policymakers in donor agencies and international organizations could draw two quite different lessons from our findings.

First, that existing efforts to convey messages about the learning crisis have not yet fully succeeded, and so efforts should be redoubled. We found that officials who estimated lower learning levels were more likely to prioritize foundational literacy, although this relationship was only marginally statistically significant. Investment in more research that informs policy makers about the actual status of schools in their country might help correct their overestimation of learning levels and increase their support for foundational literacy. This could include simply better presentation and communication of existing research - many respondents in our sample vastly over-estimated learning levels despite there being multiple national learning assessments published in their country. We show that a majority of officials do not have a ‘growth mindset’ and believe that intelligence is fixed. This contrasts with research that shows schooling can increase intelligence, but aligns with research on the fixed mindset of many teachers on the learning potential of some students. To achieve universal foundational literacy, donors could explore ways to alter these perception.

Second, and alternatively, that developing country governments have a preference for projects focused on technical and vocational education. And so more efforts should be put into identifying and supporting effective models. Donors who are committed to principles of “country ownership” must grapple with the fact that countries have legitimate education goals beyond basic skills, e.g. jobs and political cohesion.

Our findings on evidence use support a case for more localized research. Officials are more likely to update their beliefs when presented with evidence from another low- or middle-income country. They were more likely to update their beliefs if the study had a large sample size. However, the study being an RCT had no effect on their likelihood of updating beliefs towards the true effect size of the study. We should have less confidence that the findings of a single RCT will be accepted globally. Researchers could work more closely with officials and policy makers to design studies that are more likely to convince them to update their beliefs.

There are several limitations to our analysis. Firstly, while presenting hypothetical scenarios provide controlled conditions for eliciting preferences, they lack the contextual complexities of real-world decision-making. Policy decisions in practice are shaped by high-stakes environments and are influenced by a myriad of political, social, and ideological factors that cannot be fully captured in simplified scenarios. Consequently, the preferences expressed here may only partially reflect actual policy priorities under real-world conditions.

Additionally, the structure of choice scenarios, which resembles consumer choice models, may not entirely capture the strategic considerations of policymakers, whose decisions are often affected by external pressures, stakeholder influences, and broader political economy dynamics. The study also assumes that preferences expressed in hypothetical contexts align with long-term policy goals, though real-world decisions may involve trade-offs that complicate these preferences. Lastly, the study’s focus on specific variables within hypothetical scenarios limits its ability to account for the interaction of multiple contextual factors simultaneously affecting decisions in diverse educational contexts.

Future research could address these limitations by exploring case studies or ethnographic research that examine policymakers’ preferences in situ, providing insights into the impact of real-world complexities on educational decision-making.

Future research could usefully expand to more countries, or more topics that are of particular interest to major donors, such as girl’s education. It could also expand to other officials and policy makers responsible for making and implementing policy, e.g. Members of Parliament and District Education Officers; or officials responsible for investing in social sectors, e.g. officials at the Ministry of Finance. Understanding better the knowledge, perceptions and priorities of officials and policy makers in aid-recipient countries could help donors optimize the impact of aid money.

There is more to be learnt about the conditions under which rigor and external validity play a role in how policymakers are influenced by evidence. Researchers generally want their work to have an impact in the real world. For this to happen, officials and policy makers need to be able to access research and to be convinced that it is sufficiently credible and relevant to change their minds and inform policy. While there has been an emerging literature on this topic in recent years, more rigorous work – including with officials and policy makers in low- and middle-income countries – could make research more relevant to officials and policy makers and ultimately have more impact.

## CRediT authorship contribution statement

**Lee Crawfurd:** Writing – review & editing, Writing – original draft, Formal analysis, Conceptualization. **Susannah Hares:** Writing – review & editing, Writing – original draft, Formal analysis, Conceptualization. **Ana Minardi:** Writing – review & editing, Writing – original draft, Formal analysis, Conceptualization. **Justin Sandefur:** Writing – review & editing, Writing – original draft, Formal analysis, Conceptualization.

## Declaration of competing interest

The authors have no competing interests to declare.

## Data Availability

Replication data is available at the CGD dataverse: https://dataverse.harvard.edu/dataverse/cgdev.

## Appendix A Supplementary data

MMC S1 Supplementary materials: additional figures, tables, and robustness checks.

## Footnotes

1 The World Bank’s flagship 2018 World Development Report focused on the ‘Global Learning Crisis’. This report defines a global learning crisis as severe shortfalls in learning, e.g., that less than half of children in Africa who make it to the last year primary school achieve basic minimum levels of functional literacy and numeracy. In the same year, the UK government’s aid ministry outlined a new education policy with the objective of ‘tackling the learning crisis at its root’ (Department for International Development, 2018), and a new USAID education policy repeated ‘There is a learning crisis’ (United States Agency for International Development, 2018). The learning crisis was the focus of the Bill and Melinda Gates Foundation’s global education program strategy (Bill and Melinda Gates Foundation, 2021), and the 2016 Education Commission report (The International Commission on Financing Global Education Opportunity, 2016). The new focus is apparent in the growth of the phrase ‘learning crisis’ across academic literature (Figure A1), and is reflected in spending patterns — among large donors to education, the United States and United Kingdom have by the far the highest spending on primary education (64 percent and 40 percent of all their education aid, respectively), and amongst the lowest spending on vocational education (2 percent and 7 percent, respectively. Figure A2).

2 We compare the characteristics of respondents from the original sample and the additional respondents in Table B1. Additional respondents are somewhat less likely to be at the Ministry of Education (57 percent, vs 70 percent in the original sample), and less likely to be a director or assistant director (44 percent, vs 60 percent in the original sample. We also show that our main findings are robust to focusing only on those respondents from the original sample in Appendix C.

3 Donors spend large sums on advice and technical assistance for partner governments. For example the World Bank alone spends on the order of $200 million per year on providing advice to developing countries (Knack et al., 2020). Total technical assistance from DAC donors is 6 percent of all bilateral aid, or around $4 billion per year (OECD, 2017). Given wide-ranging uncertainties in the policy-making process, it is very hard to quantitatively assess the value provided by technical assistance. Advice might be high quality but fail to be of use due to unforeseen political or administrative constraints, which may or may not be the fault of the advisor.

4 The World Bank provides estimates of learning poverty for 14 of the countries in our sample, and estimates of harmonized learning outcomes for another 19 countries. We have no comparable data for Comoros, Cote D’Ivore, and DR Congo. For the 19 countries with harmonized learning outcome data but no learning poverty data, we impute learning poverty using a simple bivariate regression. In this we regress learning poverty on harmonized learning outcomes for the 113 countries with data (as of 2020). The R-squared in this regression is high: 0.785.

5 Specifically, we construct three indicator variables for each question, indicating whether the individual rated that item as their first, second, or third priority. We then calculate a principal components index of all indicator variables, and standardize this index to mean zero, standard deviation one.
