# Supplementary material for: Understanding education policy preferences: Survey experiments with policymakers in 35 developing countries
Source: World Dev. 2025 Dec;196:107140. doi: 10.1016/j.worlddev.2025.107140 (PMC12549476; doi:10.1016/j.worlddev.2025.107140)
Supplement: MMC S1 — Supplementary materials: additional figures, tables, and robustness checks. [file mmc1.pdf]

A Appendix: Additional Figures

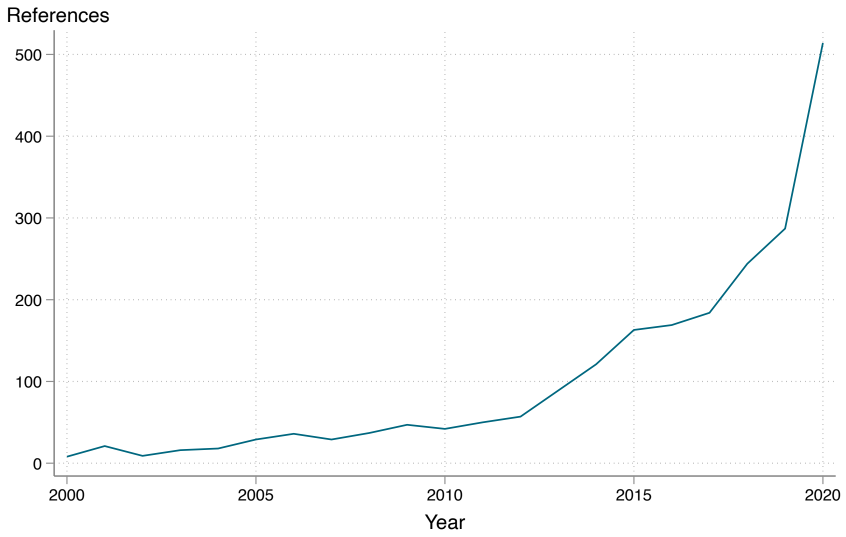

Figure A1: Academic publications per year mentioning ‘Learning crisis’

Note: Search results from Google Scholar, per year.

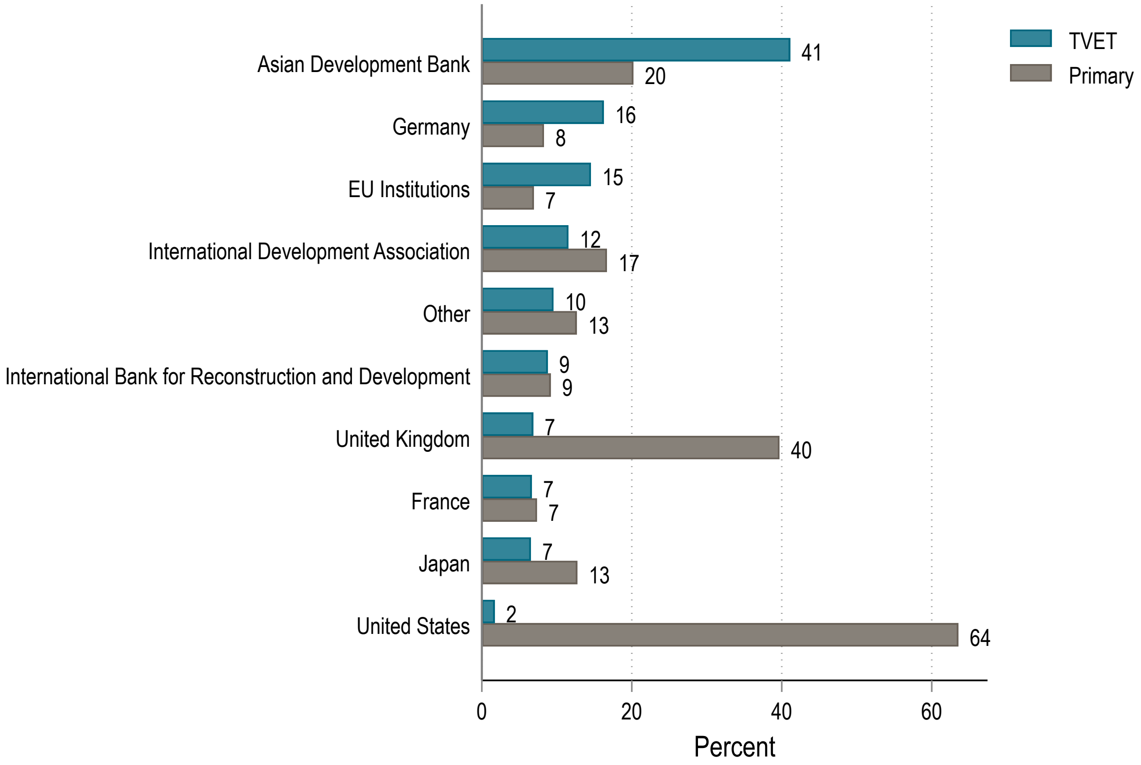

.

Figure A2: Aid Spending Varies Significantly

Note: This figure shows the share of all spending on education aid that goes on technical or vocational education (TVET), at either secondary or tertiary level, or on primary schooling, by donor. The nine largest donors to education are shown independently, with all other donors included in the "Other" category. Data is from the 2019 OECD Creditor Reporting System (CRS) database.

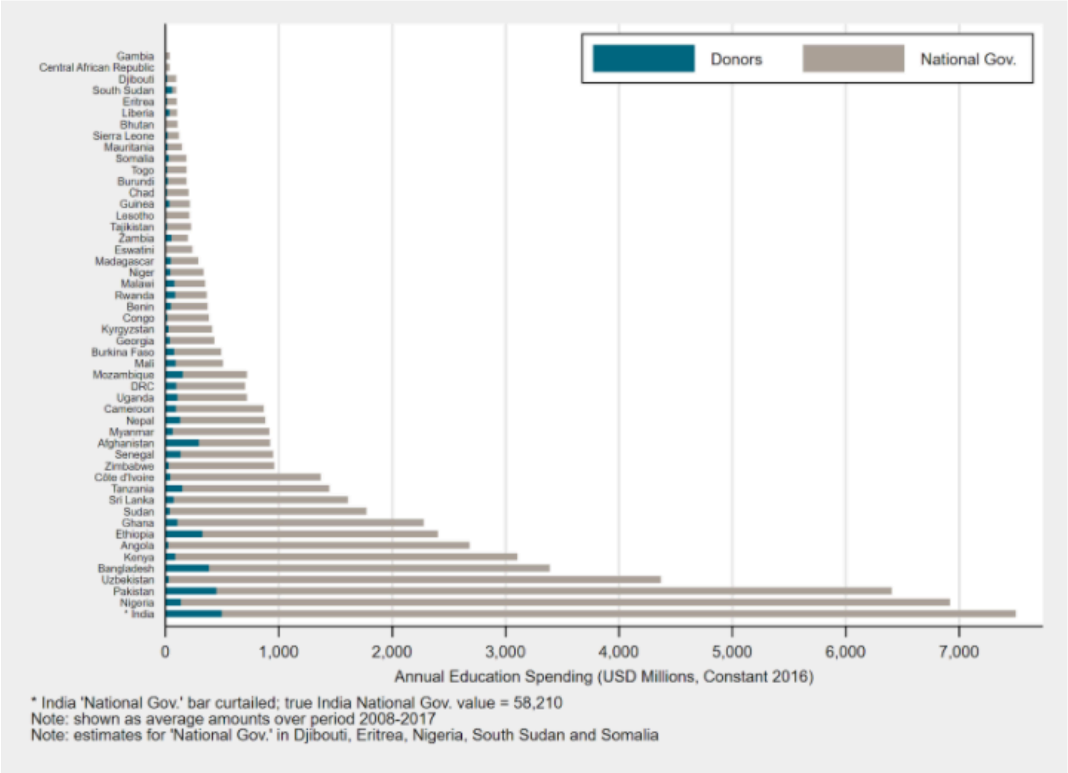

Figure A3: Aid is a low share of all public spending on education

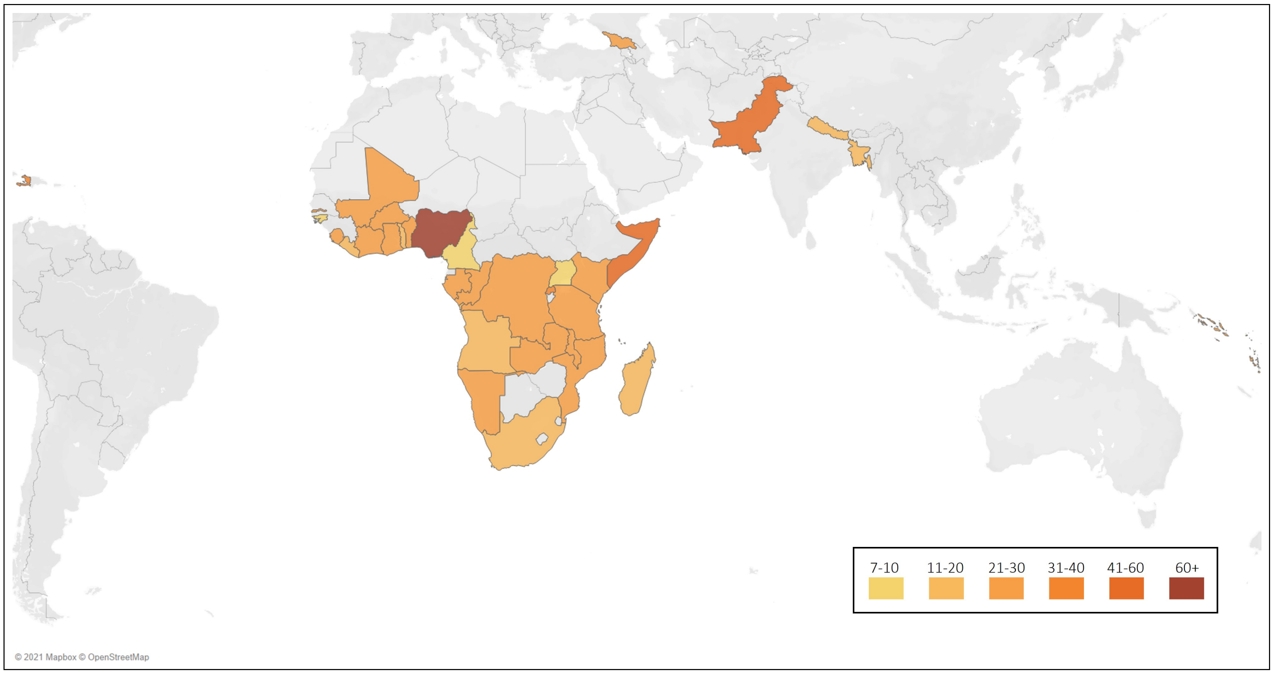

Figure A4: Country sample size map

.

Note: The full list of sampled countries is Gambia, Liberia, Malawi, Sierra Leone, Tanzania, Uganda, Benin, Burkina Faso, DRC, Madagascar, Mali, Rwanda, Togo, Nepal, Guinea-Bissau, Haiti, Mozambique, Somalia, Ghana, Kenya, Nigeria, Zambia, Cameroon, Comoros, Congo, Cote d'Ivoire, Bangladesh, Pakistan, Solomon Islands, Vanuatu, Angola, Namibia, South Africa, Gabon, and Georgia

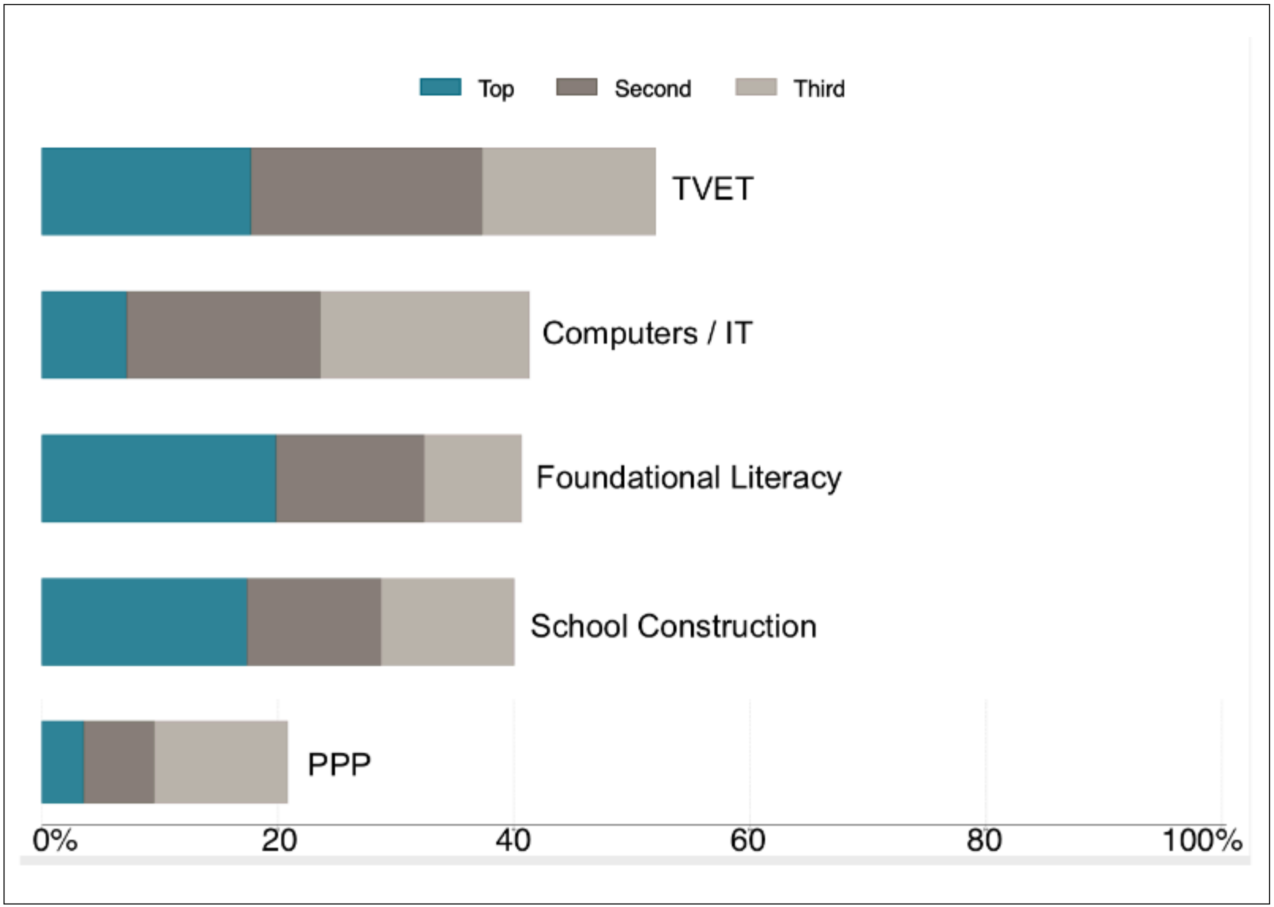

Figure A5: Stated policy priorities

Note: The question asked was: *What would your priorities be for any new additional aid spending? Please select your top 3*

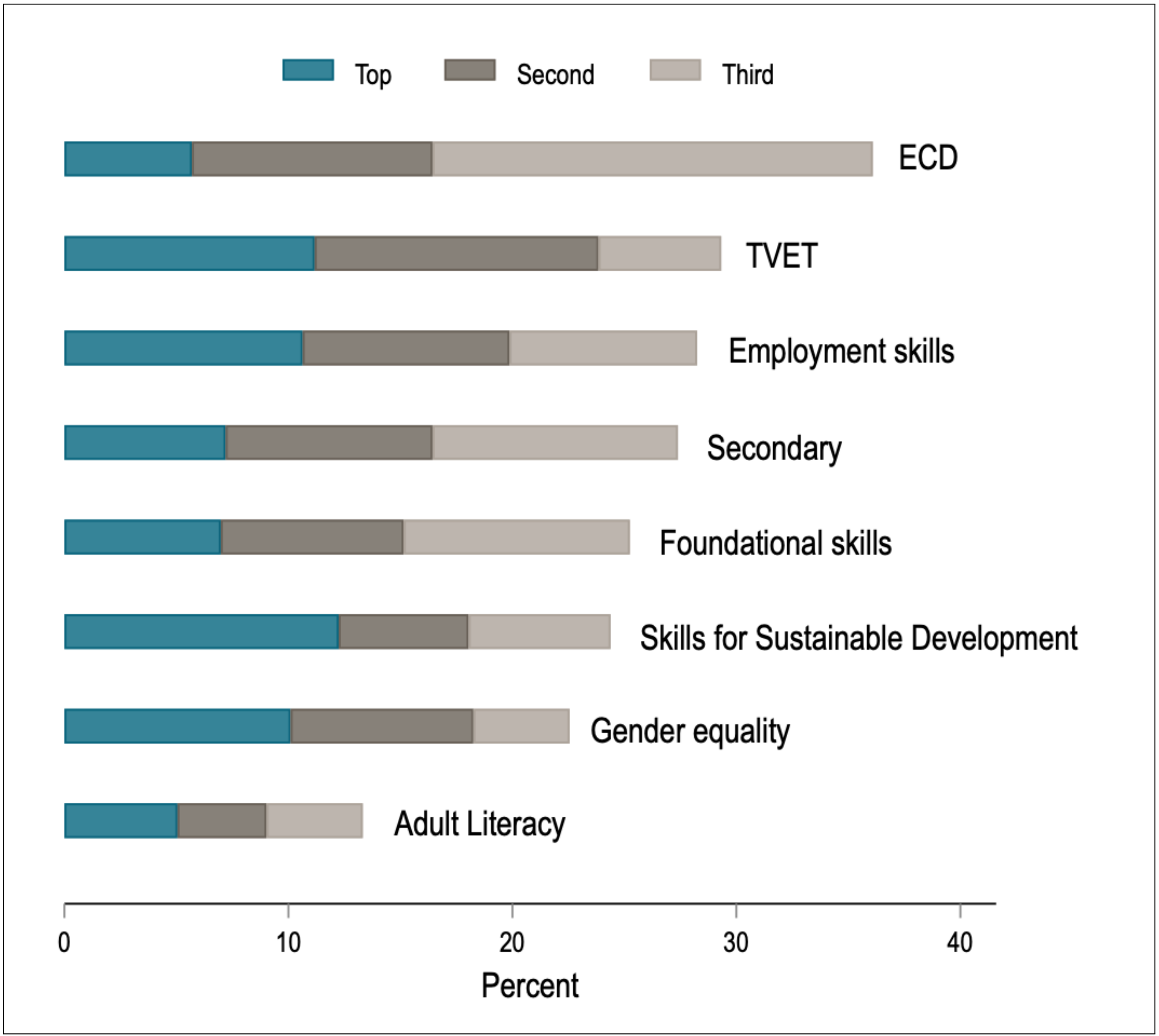

Figure A6: Which are the three most important Sustainable Development Goals?

Note: There are several targets associated with the United Nations Sustainable Development Goal on Education (SDG 4). Which 3 are most important for your country?

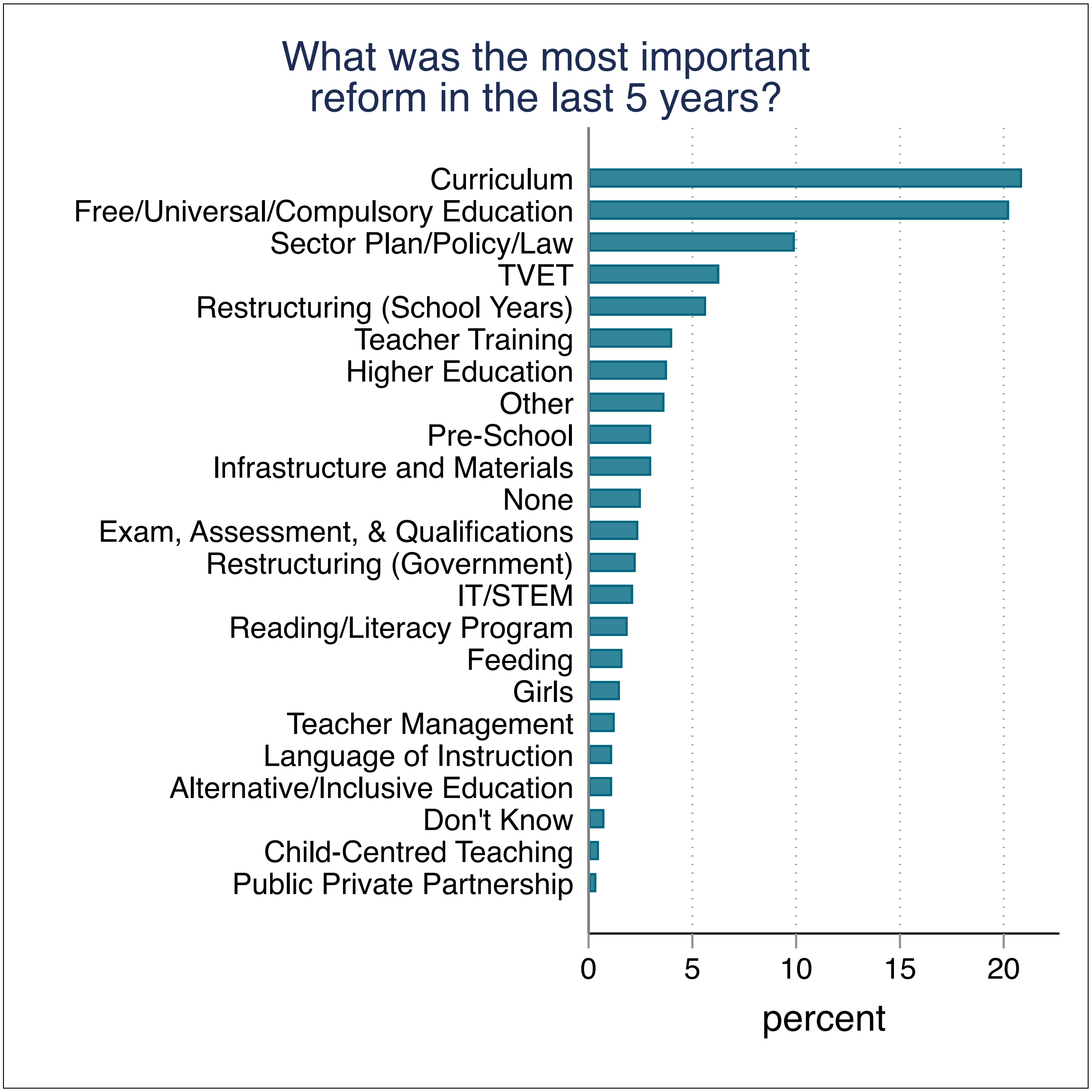

Figure A7: Reforms officials view as most important

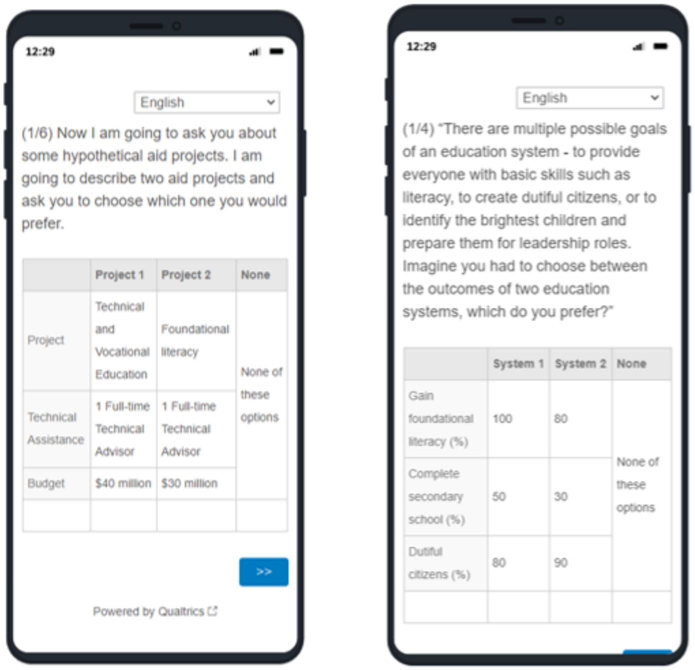

Figure A8: Discrete choice experiment - example screen

Note: The values shown here are illustrative examples. The features of each project and system are randomly generated for each choice. Each official faces six randomly generated choices. In each case, the project can be either a) School construction, b) Foundational literacy, c) Learning Assessment, d) Computers / Technology, or e) Technical and Vocational Education. The Technical Assistance can consist of a) None, b) 1 Full-time Technical Advisor, or c) 2 Full-time Technical Advisors. The Budget can consist of $30 million, $32 million, $35 million, $37 million, or $40 million.

B Appendix: Additional Tables

Table B1: Balance test on shortlisted and additional respondents

	(1) Additional		(2) Original list		T-test Difference
Variable	N	Mean/SE	N	Mean/SE	(1)-(2)
Male	235	0.71	680	0.74	-0.03
		(0.03)		(0.02)	
Years of experience	216	13.33	663	14.57	-1.24
		(0.69)		(0.46)	
Agency is Ministry of Education	248	0.56	684	0.70	-0.14***
		(0.03)		(0.02)	
Is a director or an assistant/deputy director	248	0.45	684	0.60	-0.15***
		(0.03)		(0.02)	

Notes: The value displayed for t-tests are the differences in the means across the groups. ***, **, and * indicate significance at the 1, 5, and 10 percent critical level.

Table B2: Explaining Policy Preferences

	Foundational	TVET	Empl	Skills	Adult	Gender	ECD	USE
Beliefs about reality:								
Foundational Learning	-0.0311	0.0496*	0.0346	0.0283*	0.000694	0.00470	0.0491**	0.0431**
	(0.0187)	(0.0255)	(0.0234)	(0.0146)	(0.0169)	(0.0183)	(0.0219)	(0.0161)
Average schooling (years)	0.000989	0.00143	-0.00229	-0.00357	-0.000649	-0.0103	0.00274	0.00665
	(0.00630)	(0.00604)	(0.00861)	(0.00743)	(0.00367)	(0.00739)	(0.00775)	(0.00729)
LM Returns (z-score)	0.0188	0.0187	-0.0170	0.0220	0.00529	-0.0252*	0.00189	-0.0425**
	(0.0157)	(0.0241)	(0.0179)	(0.0187)	(0.0113)	(0.0135)	(0.0162)	(0.0190)
Growth Mindset (1-6 score)	0.000648	0.00529	0.00189	0.0382***	-0.00902	0.0156	0.0204	0.0141*
	(0.0116)	(0.0147)	(0.0170)	(0.0135)	(0.00945)	(0.0150)	(0.0143)	(0.00781)
Beliefs about interventions:								
Scripted lessons (0-4 scale)	0.0103	-0.00982	-0.0146	-0.0186	-0.00217	0.0130	0.0176	-0.0409**
	(0.0275)	(0.0227)	(0.0214)	(0.0154)	(0.0127)	(0.0231)	(0.0312)	(0.0183)
Respondent Characteristics:								
Male	-0.0808**	-0.0312	0.0361	0.0234	-0.0292	-0.0555*	0.0211	-0.0175
	(0.0350)	(0.0362)	(0.0494)	(0.0354)	(0.0290)	(0.0298)	(0.0293)	(0.0262)
TVET Agency	-0.0543	0.171*	0.295***	0.0764	0.0370	0.0485	-0.167**	0.0899
	(0.0784)	(0.0907)	(0.0737)	(0.0556)	(0.0573)	(0.0728)	(0.0727)	(0.0759)
Country Characteristics:								
Share of aid on TVET	-0.0783	-0.224	-0.224	0.574**	-0.171	-0.573	0.264	0.577**
	(0.244)	(0.353)	(0.416)	(0.255)	(0.243)	(0.341)	(0.254)	(0.241)
Grad unemployment	-0.0144**	-0.0110*	-0.00895	-0.0148**	-0.00453	-0.0151**	-0.0191**	-0.0126**
	(0.00554)	(0.00593)	(0.00684)	(0.00584)	(0.00354)	(0.00608)	(0.00824)	(0.00513)
Region FE	Yes	Yes	Yes	Yes	Yes	Yes	Yes	Yes
Outcome Mean	0.25	0.26	0.28	0.26	0.12	0.22	0.34	0.27
Obs.	680	680	680	680	680	680	680	680
R^2	0.05	0.05	0.08	0.09	0.02	0.08	0.11	0.13

Note: The outcome variable is a binary indicator taking the value 0 or 1 for whether the respondent chose each Sustainable Development Goal as one of the top three priorities for their country. The seven Sustainable Development Goals in education cover technical and vocational education (TVET), employment skills, skills for sustainable development, adult literacy, gender equality, early child development services (ECD), or universal secondary education (USE). In addition these seven, we offered respondents the option to select foundational skills, which is one of the sub-indicators for the goals. Standard errors are clustered at the country level.

* p<0.1, ** p<0.05, *** p<0.01

Table B3: Explaining Aid Spending Preferences

	Foundational	TVET	Construction	PPP	Computers
Beliefs about reality:					
Foundational Learning	0.00223	0.0121	0.0435	0.00892	0.0405
	(0.0341)	(0.0310)	(0.0277)	(0.0194)	(0.0316)
Average schooling (years)	-0.00423	0.000533	0.00981	-0.00518	0.00357
	(0.00892)	(0.00995)	(0.00971)	(0.00461)	(0.0100)
LM Returns (z-score)	-0.0217	0.0398*	0.0121	0.0106	-0.0212
	(0.0266)	(0.0226)	(0.0199)	(0.0141)	(0.0218)
Growth Mindset (1-6 score)	0.0308	0.00898	0.00594	0.0179	0.0199
	(0.0213)	(0.0231)	(0.0158)	(0.0115)	(0.0216)
Beliefs about interventions:					
Scripted lessons (0-4 scale)	0.0156	-0.0276	0.00364	-0.0102	-0.0179
	(0.0248)	(0.0270)	(0.0326)	(0.0257)	(0.0263)
Respondent Characteristics:					
Male	-0.0813**	0.0159	-0.0487	0.0559	-0.0495
	(0.0307)	(0.0355)	(0.0348)	(0.0361)	(0.0371)
TVET Agency	-0.0247	0.210**	0.0259	0.120**	0.250***
	(0.0869)	(0.0901)	(0.0823)	(0.0507)	(0.0615)
Country Characteristics:					
Share of aid on TVET	0.119	0.345	0.525	-0.0786	0.0302
	(0.373)	(0.467)	(0.347)	(0.250)	(0.347)
Grad unemployment	-0.0177**	-0.0209*	-0.0267***	-0.000443	-0.0136
	(0.00834)	(0.0111)	(0.00829)	(0.00408)	(0.00816)
Region FE	Yes	Yes	Yes	Yes	Yes
Outcome Mean	0.41	0.51	0.40	0.20	0.43
Obs.	680	680	680	680	680
R^2	0.06	0.10	0.11	0.04	0.08

Note: The outcome variable is an index summarising the strength of individual preference for foundational learning or for TVET. Standard errors are clustered at the country level.
* p<0.1, ** p<0.05, *** p<0.01

Table B4: Conjoint Experiment: Aid Project Preferences (Logit Model)

	Full Sample	Full Sample	TVET Ministry/ Agency	Other Ministries & agencies
choice				
Budget (USD million)	0.0469***	0.0499***	0.0203	0.0552***
	(0.00670)	(0.00714)	(0.0282)	(0.00753)
Technical Advisors	0.147***	0.156***	0.385***	0.129***
	(0.0290)	(0.0306)	(0.135)	(0.0320)
TVET	0.495***	0.523***	1.185***	0.466***
	(0.0713)	(0.0756)	(0.276)	(0.0804)
Assessment	0.0848	0.0918	0.536*	0.0272
	(0.0709)	(0.0747)	(0.285)	(0.0795)
Foundational Literacy	-0.0238	-0.0218	0.225	-0.0310
	(0.0709)	(0.0747)	(0.303)	(0.0788)
School Construction	0.0838	0.0902	0.830***	0.0256
	(0.0689)	(0.0727)	(0.291)	(0.0769)
Resp. FE		Yes	Yes	Yes
Obs. (Responses)	8,558	8,558	568	7,676
Pseudo R^2	.0114195	.0120764	.0431561	.0118294

Note: Coefficients for TVET, assessment, foundational literacy, and school construction projects are also compared to the omitted reference category of an IT project. Columns 2-4 include respondent fixed effects. Standard errors, clustered at the paired comparison level, are in parentheses. * $p < 0.1$, ** $p < 0.05$, *** $p < 0.01$. Standard errors in parentheses.

Table B5: Conjoint Experiment: Aid Project Preferences (Marginal Effects of Logit Model)

	Full Sample	Full Sample	TVET Ministry/ Agency	Other Ministries & agencies
Budget (USD million)	0.0115***	0.0123***	0.0048	0.0136***
	(0.0016)	(0.0017)	(0.0066)	(0.0018)
Technical Advisors	0.0361***	0.0384***	0.0907***	0.0317***
	(0.0071)	(0.0075)	(0.0308)	(0.0078)
TVET	0.1219***	0.1287***	0.2788***	0.1146***
	(0.0173)	(0.0184)	(0.0610)	(0.0196)
Assessment	0.0209	0.0226	0.1261*	0.0067
	(0.0174)	(0.0184)	(0.0664)	(0.0196)
Foundational Literacy	-0.0059	-0.0054	0.0529	-0.0076
	(0.0175)	(0.0184)	(0.0711)	(0.0194)
School Construction	0.0206	0.0222	0.1954***	0.0063
	(0.0170)	(0.0179)	(0.0663)	(0.0189)
Obs. (Responses)	8,558	8,558	568	7,676

Note: Coefficients for TVET, assessment, foundational literacy, and school construction projects are also compared to the omitted reference category of an IT project. Columns 2-4 include respondent fixed effects. Standard errors, clustered at the paired comparison level, are in parentheses. * $p < 0.1$, ** $p < 0.05$, *** $p < 0.01$. Standard errors in parentheses.

Table B6: Conjoint Experiment: Education Outcome Preferences (Logit Model)

	Full Sample	Full Sample	TVET Ministry/ Agency	Other Ministries & agencies
choice				
+10% point with FL	0.279***	0.300***	0.203***	0.314***
	(0.0170)	(0.0185)	(0.0631)	(0.0198)
+10% point complete secondary	0.325***	0.352***	0.401***	0.349***
	(0.0176)	(0.0193)	(0.0704)	(0.0206)
+10% point are dutiful citizens	0.417***	0.449***	0.385***	0.447***
	(0.0251)	(0.0269)	(0.0929)	(0.0287)
Resp. FE		Yes	Yes	Yes
Obs. (Responses)	6,730	6,730	542	5,942
Obs. (Respondents)	3,365	3,365	271	2,971
Pseudo R^2	0.104	0.112	0.106	0.113

Note: Columns 3-4 include respondent fixed effects. Standard errors, clustered at the paired comparison level, are in parentheses.

* $p < 0.1$, ** $p < 0.05$, *** $p < 0.01$. Standard errors in parentheses.

Table B7: Conjoint Experiment: Education Outcome Preferences (Marginal Effects of Logit Model)

	Full Sample	Full Sample	TVET Ministry/ Agency	Other Ministries & agencies
+10% point with FL	0.0602***	0.0640***	0.0436***	0.0669***
	(0.0034)	(0.0037)	(0.0131)	(0.0039)
+10% point complete secondary	0.0700***	0.0752***	0.0862***	0.0742***
	(0.0034)	(0.0037)	(0.0130)	(0.0039)
+10% point are dutiful citizens	0.0899***	0.0959***	0.0829***	0.0951***
	(0.0050)	(0.0053)	(0.0188)	(0.0057)
Obs. (Responses)	6,730	6,730	542	5,942

Note: Standard errors, clustered at the paired comparison level, are in parentheses.

* $p < 0.1$, ** $p < 0.05$, *** $p < 0.01$. Standard errors in parentheses.

C Appendix: Results omitting additional sample

As discussed in section 2, our initial sample frame contained 1,056 potential respondents, of whom 684 were successfully interviewed (65 percent). An additional 247 interviews were conducted with respondents who were not on the initial lists, but did meet the criteria for interview, for a total of 931 interviews. In this Appendix we show that our main results are robust to omitting the additional 247.

Table C1: Beliefs about Reality

	Mean(Beliefs)	Mean(Data)	SE(Beliefs)	N(Beliefs)	N(Data)
Global learning crisis (0/1)	.75	.	.43	439	.
National learning crisis (0/1)	.79	.	.41	439	.
10yr olds can read	50.32	27.92	23.78	325	24
Average schooling (Years)	10.45	8.95	3.64	382	24
Gov spend per child (USD)	182.35	183.92	187.79	289	24
Growth mindset (1-6)	3.84	.	1.67	422	.
LM Returns for Boys	90.17	59.51	75.86	186	17
LM Returns for Girls	111.59	77.77	91.85	82	18
Effect of Reading Program (0-4)	2.23	2.53	1.01	413	24

Notes: Data on reading comes from the World Bank reading poverty indicator, and for schooling and spending from the World Bank Development Indicators. Data for labour market returns are drawn from Montenegro and Patrinos (2014). The effects of three reading programs described to respondents come from Cilliers et al. (2016), Jackson and Makarin (2018), and Piper et al. (2018). Further detail about the estimates of labour market returns is contained in Annex C and Table D1.

Table C2: Conjoint Experiment: Aid Project Preferences

	Full Sample	Full Sample	TVET Ministry/ Agency	Other Ministries & agencies
Budget (USD million)	0.0122***	0.0122***	-0.00203	0.0133***
	(0.00195)	(0.00195)	(0.00685)	(0.00205)
Technical Advisors	0.0402***	0.0402***	0.0522	0.0367***
	(0.00835)	(0.00835)	(0.0317)	(0.00872)
TVET	0.105***	0.105***	0.245***	0.0937***
	(0.0206)	(0.0206)	(0.0615)	(0.0218)
Assessment	0.00747	0.00747	0.139**	-0.00599
	(0.0206)	(0.0206)	(0.0662)	(0.0219)
Foundational Literacy	-0.0219	-0.0219	0.0318	-0.0235
	(0.0208)	(0.0208)	(0.0698)	(0.0218)
School Construction	0.00871	0.00871	0.174**	-0.00739
	(0.0202)	(0.0202)	(0.0672)	(0.0212)
Respondent FE	Yes	Yes	Yes	Yes
Obs. (Responses)	7,008	7,008	552	6,384
Obs. (Respondents)	3,504	3,504	276	3,192
R^2	0.058	0.058	0.159	0.051

Note: The unit of observation is a hypothetical aid project presented to an individual respondent, and the dependent variable is an indicator that the project was selected as preferable (from a set of two options). Estimates are based on a linear probability model. Results are similar using a logit model (Table B4 and Table B5). The omitted category for projects is an IT project. Results are similar when estimating marginal means rather than average marginal component effects (Leeper et al., 2020). All specifications include respondent fixed effects. Standard errors, clustered at the paired comparison level, are in parentheses

* p<0.1, ** p<0.05, *** p<0.01

Table C3: Conjoint Experiment: Education Outcomes Preferences

	Full Sample	TVET Ministry/ Agency	Other Ministries & agencies
+10% point with FL	6.517***	3.330**	6.890***
	(0.484)	(1.576)	(0.512)
+10% point complete secondary	8.411***	9.946***	8.257***
	(0.490)	(1.640)	(0.517)
+10% point are dutiful citizens	9.831***	8.813***	9.845***
	(0.699)	(2.329)	(0.738)
Obs. (Responses)	4,878	456	4,366
Obs. (Respondents)	617	57	553
R^2	0.155	0.153	0.158

Note: The unit of observation is a hypothetical state of the world, presented to an individual respondent. The dependent variable is an indicator that this state of the world was preferred (from a set of two options). Estimates are based on a linear probability model. Results are similar using a logit model (Table B6 and Table B7). All specifications include respondent fixed effects. Standard errors, clustered at the paired comparison, are in parentheses

* p<0.1, ** p<0.05, *** p<0.01

Table C4: Explaining Spending Preferences

	FLN		TVET	
	(1)	(2)	(3)	(4)
Beliefs about reality:				
Foundational Learning	-0.0928	-0.0584	0.138*	0.172**
	(0.0549)	(0.0533)	(0.0674)	(0.0703)
Average schooling (years)	-0.00681	0.00133	-0.00685	-0.0228
	(0.0183)	(0.0200)	(0.0217)	(0.0222)
LM Returns (z-score)	0.0136	0.0151	0.0221	0.0357
	(0.0387)	(0.0451)	(0.0587)	(0.0589)
Growth Mindset (1-6 score)	0.00400	0.00913	0.0364	0.0446
	(0.0362)	(0.0340)	(0.0595)	(0.0512)
Beliefs about interventions:				
Scripted lessons (0-4 scale)	0.104	0.0621	-0.0862	-0.0660
	(0.0668)	(0.0790)	(0.0756)	(0.0528)
Respondent Characteristics:				
Male		-0.107		0.0265
		(0.0713)		(0.0942)
TVET Agency		-0.130		0.811***
		(0.230)		(0.216)
Country Characteristics:				
Share of aid on TVET		2.070*		2.566
		(1.155)		(1.987)
Grad unemployment		-0.0395**		-0.0439*
		(0.0185)		(0.0236)
Region FE	Yes	Yes	Yes	Yes
Obs.	594	498	594	498
R^2	0.03	0.08	0.04	0.14

Note: The outcome variable is an index summarising the strength of individual preference for foundational learning or for TVET. We show results for the individual components of this index in Table B2 and Table B3. Standard errors are clustered at the country level.
* p<0.1, ** p<0.05, *** p<0.01

D Appendix: Understanding Policymaker beliefs about the returns to school

What explains variation in policymaker beliefs about the returns to school? We first calculate the expected returns ($Returns_k$) for each hypothetical child, as the percentage growth in earnings from completing secondary school. This is the difference between expected earnings with secondary and expected earnings with primary, as a percentage of expected earnings with primary. The average estimated return to secondary school is 98 percent.

We then regress this measure of expected returns on the characteristics of the hypothetical child, and characteristics of the official responding. As there are four observations per respondent, we cluster standard errors by individual respondent.

$$Returns_k = \sum_{k=1}^{3} \beta_k X_k + \sum_{j=1}^{3} \delta_j Z_j + \epsilon \tag{4}$$

Where X_k are characteristics of the hypothetical child, such as being a girl or a boy, poor or rich and have a high or low IQ. We also include respondent characteristics as controls, represented by Z_j which includes gender, years of experience and their office role.

Contrary to our hypothesis, there is no statistically significant difference in perceived returns to education for girls and boys, or for low or high intelligence children. Officials expect returns to be 15 percentage points higher for children from poor families than from rich families. Female officials have higher expectations than male officials, particularly for girls (Table D1). We also see no correlation between the growth mindset of the respondent and the degree to which they think that initial intelligence matters for the labour market returns to education.

Table D1: Correlates of beliefs about returns to education

	(1)	(2)	(3)	(4)
Child: Girl (vs Boy)	6.772	8.570	-2.396	4.912
	(8.135)	(7.758)	(7.910)	(7.297)
Child: Poor Family (vs Rich Family)	15.450***	15.487***	15.416***	15.881***
	(3.604)	(3.583)	(3.590)	(3.609)
Child: High IQ (vs Low IQ)	3.077	3.200	3.240	7.367
	(3.101)	(3.117)	(3.117)	(8.150)
Official: Female		20.431***	9.784	16.445**
		(7.794)	(7.859)	(7.255)
Official: Female X Child: Girl			33.925*	
			(20.014)	
Official: Growth Mindset				-2.066
				(2.469)
Official: Growth Mindset X Child: High IQ				-1.250
				(2.070)
Controls	No	Yes	Yes	Yes
Outcome Mean	92.660	92.660	92.660	92.049
N (Responses)	2,005	2,005	2,005	1,984
N (Respondents)	527	527	527	521
R^2	0.007	0.052	0.058	0.056

Note: The outcome is the official's belief about the labour market return to secondary school over primary school. Controls include official's experience, job category, agency, government level (national or sub-national), and world region.

* $p < 0.1$, ** $p < 0.05$, *** $p < 0.01$. Standard errors in parentheses.

E Appendix: Ethics

We did not explicitly ask participants for informed consent in the research, in line with APSA guidelines on human subjects research which allows an exception where respondents are powerful actors and institutions. We expected there to be no harm from participation, beyond their voluntary time commitment from a single interview. We took care to ensure that individuals were not individually identifiable from the anonymised publicly released survey data. We also sought to minimise the length of time of the interview. No deception was used in the research. The researchers do not work at institutions that require approval from an institutional review board (IRB), however we did take ethics seriously.

We informed participants of the purpose of the research - specifically the following preamble was read:

> "This is a survey on international education from [Organisation-redacted].
> [Detail about redacted organisation]. Your response will be of great value in informing major donors and international organisations about the view from government in developing countries.
> All responses will remain anonymous."

Fieldwork was conducted during the first wave of the global covid pandemic in 2020, and so we designed the survey to be able to be administered either face to face, or where that was not possible remotely by phone, video call, or email, to allow for social distancing. Each country was though at different stages of covid outbreak and had different restrictions in place, so we allowed survey consultants in each country to use their own judgement in order to ensure they followed local guidance and stayed safe.

We did not anticipate any political impacts from this research.

We offered to share the final research paper with any interested participant, and have sent a draft of this paper by email to all who were interested.
